# Comparative Evaluation of Changes in Brain Activity Among Edentulous Patients Rehabilitated With Conventional Complete Dentures and Tooth-Supported Overdentures Using Electroencephalography: An In Vivo Study

**DOI:** 10.7759/cureus.113870

**Published:** 2026-08-03

**Authors:** Shabir Ahmed Shah, Schubert Anto R., Mansi Manohar Gadhe, Adiba Ihsan, Shazia Kosar

**Affiliations:** 1 Department of Prosthodontics and Crown and Bridge, Government Dental College and Hospital, Srinagar, IND

**Keywords:** complete, dentulous mouth, denture, electroencephalography, mastication, overdenture

## Abstract

Introduction: Edentulism is associated with impaired masticatory function and reduced sensory stimulation that may adversely affect cortical activity and cognitive health. However, evidence comparing the effects of different removable prosthetic rehabilitation modalities on brain activity is limited. Our study aimed to evaluate and compare changes in brain activity using electroencephalography among completely edentulous patients rehabilitated with conventional complete dentures, tooth-supported overdentures without attachments, and tooth-supported overdentures with stud attachments.

Materials and methods: This prospective observational study included 36 participants aged 60-80 years who fulfilled the eligibility criteria. The participants received routine prosthodontic treatment based on their clinical condition, were non-randomized, and were categorized into three groups (n = 12 each): conventional complete dentures, tooth-supported overdentures without attachments, and tooth-supported overdentures with stud attachments. Baseline electroencephalographic recordings were obtained before treatment and repeated after two months of prosthesis use under standardized recording conditions. Baseline demographic characteristics were compared using one-way analysis of variance (ANOVA) and the chi-square test. Changes in brain activity over time were evaluated using a mixed-design repeated-measures ANOVA, with supplementary independent-samples and paired-samples t-tests for pairwise and within-group comparisons. Statistical significance was set at p < 0.05.

Results: The three groups were comparable in terms of age and sex at baseline. Significant changes were noticed in the measured electroencephalographic parameter after prosthodontic rehabilitation in all three groups (p ≤ 0.001). The observed increase in the EEG-derived measure following prosthodontic rehabilitation is interpreted as reflecting enhanced cortical electrical activity associated with restoration of masticatory function. However, because EEG provides an indirect measure of neural activity, these findings should not be interpreted as definitive evidence of improved cortical or cognitive function without complementary neuropsychological or functional assessments. Mixed-design repeated-measures analysis demonstrated a significant effect of time (p < 0.001), indicating that cortical activity increased following rehabilitation, irrespective of prosthesis type. Although the tooth-supported overdenture with stud attachment group demonstrated the greatest numerical increase in brain activity, neither the overall group effect nor the group-by-time interaction was significant (p > 0.05). Pairwise intergroup comparisons at baseline and after two months also revealed no significant differences.

Conclusion: Prosthodontic rehabilitation was associated with a significant increase in EEG-derived cortical brain activity after two months of functional use. Although attachment-retained tooth-supported overdentures showed the highest numerical increase, they were not statistically superior to the other treatment modalities. These findings suggest that restoration of masticatory function is associated with enhanced cortical electrical activity, irrespective of prosthesis design.

## Introduction

Edentulism remains a significant global public health concern, particularly among the aging population, and it is associated with substantial functional, psychological, and systemic consequences. Complete tooth loss has increasingly been linked to cognitive decline and impaired neurological function, beyond compromising mastication, speech, facial esthetics, and quality of life [1,2]. Recent evidence suggests that reduction in masticatory efficiency following tooth loss decreases sensory input transmitted through the trigeminal nerve, which plays an essential role in maintaining normal cortical and hippocampal activity [3]. Diminished sensory stimulation may contribute to alterations in memory, learning, and other cognitive processes, thereby increasing the risk of age-related neurodegenerative disorders.

Restoration of oral function through prosthodontic rehabilitation has the potential to reverse some of these adverse effects by improving masticatory performance and enhancing neuromuscular stimulation [4,5]. Conventional complete dentures remain the most widely used treatment modality for completely edentulous patients; however, compromised retention and stability may limit their functional efficiency [6]. In contrast, tooth-supported overdentures preserve natural tooth proprioception, improve prosthesis stability, and enhance masticatory performance, whereas the incorporation of attachments may further optimize retention and patient satisfaction [7]. These biomechanical advantages may promote greater cortical activation through improved sensory feedback during mastication.

Electroencephalography (EEG) is a non-invasive, reliable, and objective technique for recording the electrical activity of the brain and has been increasingly employed to investigate the relationship between oral rehabilitation and neural function [8]. Despite the growing interest in the association between mastication and cognition, comparative evidence evaluating the effects of different complete denture rehabilitation modalities on brain activity remains limited. Therefore, understanding whether conventional complete dentures and tooth-supported overdentures produce differential neurophysiological responses may provide valuable insights into selecting the optimal prosthodontic treatment for elderly edentulous patients.

This study aimed to compare the changes in the EEG-recorded cortical electrical activity of completely edentulous patients rehabilitated with conventional complete dentures, tooth-supported overdentures without attachments, and tooth-supported overdentures with attachments using EEG. The secondary objectives were to compare baseline and two-month post-rehabilitation EEG-recorded cortical electrical activity within each treatment group and to determine whether attachment-retained tooth-supported overdentures demonstrated greater neurophysiological changes than conventional complete dentures and non-attachment tooth-supported overdentures.

## Materials and methods

Study design and setting

This prospective observational study was conducted in the Department of Prosthodontics and Crown and Bridge, Government Dental College and Hospital, Srinagar, India, between September 2024 and December 2025. The evaluated treatment modalities represented routine prosthodontic treatment options selected according to each patient's clinical presentation and treatment requirements. Therefore, the participants were not randomized, and no allocation concealment or blinding procedures were performed, as the study was observational rather than an investigator-assigned interventional trial. Ethical approval was obtained from the Institutional Ethics Committee (GDC/Perio/eth’ committee/1767 dated July 31, 2024) before the commencement of the study, and written informed consent was obtained from all participants before enrollment.

Sample size and participant selection

The required sample size was estimated a priori using G*Power software (version 3.1; Heinrich Heine University Düsseldorf, Düsseldorf, Germany) for a repeated-measures analysis of variance (ANOVA), which was designed to compare three complete denture fabrication types for edentulous patients. Based on findings from a relevant previous study [7], an effect size of 0.55 was utilized. With a significance level of α = 0.05, a statistical power of 80%, and an equal allocation ratio among the groups, the analysis yielded a minimum total sample size of 36 participants, equating to 12 individuals for each of the three denture design groups. Consecutive patients attending the outpatient clinic who fulfilled the eligibility criteria were recruited prospectively.

The inclusion criteria comprised completely edentulous participants for the conventional denture group and participants with retained mandibular canines only for the overdenture group; an age of 60-80 years; first-time denture wearers; participants with adequate residual ridge anatomy for denture fabrication; adequate periodontal support for the retained abutment teeth; willingness to participate in the study; and the ability to attend follow-up visits at two months post-treatment. The exclusion criteria included neurological or psychiatric disorders; temporomandibular disorders (TMD); parafunctional habits (such as bruxism and clenching); xerostomia (dry mouth); excessive salivation (sialorrhea); severe systemic oral manifestations; and unfavourable residual ridge anatomy.

Baseline EEG assessment

Before prosthodontic rehabilitation, the baseline cortical activity was recorded using a digital EEG system (EEG-1200K; Nihon Kohden Corporation, Tokyo, Japan). All recordings were performed under standardized environmental conditions in a quiet room, with participants comfortably seated and instructed to remain relaxed with their eyes closed. The scalp electrodes were positioned according to the International 10-20 system to ensure reproducibility. The same neurologist performed all EEG recordings using identical parameters throughout the study to minimize operator-related variability. EEG recordings were obtained with signals recorded according to the International 10-20 electrode placement system. Resting-state EEG was recorded for five minutes with participants seated comfortably and eyes closed. The alpha frequency band (8-13 Hz) was selected as the primary outcome because it reflects resting cortical activity. Artifact-free epochs were identified after visual inspection, and spectral analysis was performed using fast Fourier transform (FFT). Mean alpha peak frequency (Hz) (or mean alpha power, if applicable) was calculated by averaging the selected electrodes and used for statistical analysis.

Prosthodontic rehabilitation procedures

All prostheses were fabricated by a single prosthodontist, following standardized clinical and laboratory procedures. Preliminary impressions were made using an impression compound (DPI Pinnacle Impression Compound; Dental Products of India Ltd., Mumbai, India), and preliminary casts were poured with type II dental plaster (Kalstone Dental Plaster; Kalabhai Karson Pvt. Ltd., Mumbai, India). The custom trays were fabricated using autopolymerizing acrylic resin (DPI-RR cold-cure acrylic resin; Dental Products of India Ltd., Mumbai, India). Border molding was completed using a green stick impression compound (DPI Pinnacle Green Stick; Dental Products of India Ltd., Mumbai, India), and definitive impressions were recorded using zinc oxide-eugenol impression paste (DPI Impression Paste; Dental Products of India Ltd., Mumbai, India). Master casts were poured into type III dental stones (Kalrock Dental Stone; Kalabhai Karson Pvt. Ltd., Mumbai, India).

In Group I, conventional complete dentures were fabricated using conventional clinical protocols. Maxillomandibular relationships were recorded using wax occlusion rims, which were mounted on a mean value articulator (Whip Mix Corporation, Louisville, KY, USA), and acrylic teeth (Acry Rock Teeth; Ruthinium Group, Badia Polesine, Italy) were arranged according to the principles of bilateral balanced occlusion. Following a satisfactory wax try-in, dentures were processed by compression molding using heat-polymerized acrylic resin (Trevalon; Dentsply Sirona, Charlotte, NC, USA) and finished, polished, inserted, and occlusally adjusted using articulating paper.

In Group II, mandibular tooth-supported overdentures without attachments were fabricated after endodontic treatment of the retained mandibular canines. Clinical crowns were reduced to approximately 2-3 mm above the gingival margin using dome-shaped preparations. Cast copings fabricated using pattern resin (Pattern Resin LS; GC Corporation, Tokyo, Japan) were cemented using conventional glass ionomer luting cement (GC Fuji I; GC Corporation, Tokyo, Japan). Impression procedures, jaw-relation records, denture processing, and insertion followed the same protocol as in Group I. Adequate relief was provided over the copings to permit passive seating while preserving periodontal proprioception.

In Group III, tooth-supported overdentures retained by stud attachments were fabricated after root canal treatment and post-space preparation of the retained canines. Cast post-core copings with ball-stud attachments (Rhein83 OT CAP Attachment System; Rhein83 S.r.l., Bologna, Italy) were fabricated using pattern resin and cemented using glass ionomer cement. During denture processing, metal housings with resilient nylon retentive caps were incorporated into the denture base using an autopolymerizing acrylic resin to provide mechanical retention. Occlusal adjustments were performed after insertion, and patients received detailed oral hygiene and maintenance instructions for both the overdenture and attachment components.

Follow-up and outcome assessment

Following prosthesis insertion, all participants underwent a two-month adaptation period to allow neuromuscular accommodation and stabilization of masticatory function. All prostheses were fabricated and delivered using a standardized clinical and laboratory protocol by the same prosthodontic team to ensure treatment consistency across all participants. Routine post-insertion adjustments were performed whenever necessary to relieve pressure areas and optimize denture retention, stability, support, and occlusal contacts until satisfactory function and patient comfort were achieved. No participant underwent major prosthesis modification or remake during the adaptation period. At the end of the follow-up period, EEG recordings were repeated under identical conditions using the same equipment, operator, electrode placement protocol, and environmental settings as the baseline examination. The primary outcome was the change in cortical brain activity from baseline to two months after prosthodontic rehabilitation. The secondary outcomes included a comparison of post-treatment EEG activity among the three rehabilitation modalities.

Statistical analysis

Data were statistically analyzed using IBM SPSS Statistics for Windows, Version 21 (Released 2012; IBM Corp., Armonk, New York, United States). Continuous variables are expressed as mean ± standard deviation, while categorical variables are summarized as frequencies and percentages. Normality of continuous variables was assessed using the Shapiro-Wilk test, and homogeneity of variances was evaluated using Levene's test. Baseline demographic characteristics were compared among the three groups using one-way ANOVA for continuous variables and the chi-square test for categorical variables. Changes in EEG brain activity over time were evaluated using a mixed-design repeated-measures ANOVA, with treatment group as the between-subjects factor and time (baseline and two months) as the within-subject factor. Main effects of group, time, and the group × time interaction were examined. Partial eta squared (ηp^2^) was calculated to estimate effect size. Where appropriate, pairwise comparisons between treatment groups at each time point were performed using independent-samples t-tests, while within-group changes from baseline to two months were evaluated using paired-samples t-tests. Two-sided p < 0.05 was considered statistically significant, and 95% confidence intervals were reported where applicable.

## Results

A total of 36 participants were included in the study, with 12 categorized into each treatment group. All participants completed the study and underwent baseline and two-month post-rehabilitation EEG assessments. Table 1 presents the baseline demographic characteristics of the participants. The mean age was comparable among the conventional complete denture group (63.78 ± 8.25 years), tooth-supported overdenture without attachment group (65.58 ± 7.25 years), and tooth-supported overdenture with attachment group (64.78 ± 7.15 years), with no statistically significant difference (p = 0.844). Similarly, the sex distribution did not differ significantly among the three groups (p = 0.717), indicating that the groups were demographically comparable at baseline.

**Table 1:** Demographic characteristics of study participants. Data are presented as mean ± standard deviation or number (percentage). Group I: conventional complete denture, Group II: overdenture with coping, Group III: overdenture with attachment; one-way analysis of variance and chi-square test were used; p < 0.05 was considered statistically significant.

Parameter	Category	Group I	Group II	Group III	Test value (df)	p-value
Age, years	Mean ± SD	63.78 ± 8.25	65.58 ± 7.25	64.78 ± 7.15	F = 0.17 (2, 33)	0.844
Sex, n (%)	Male	6 (50.0)	5 (41.7)	7 (58.3)	χ^2^ = 0.667 (2)	0.717
	Female	6 (50.0)	7 (58.3)	5 (41.7)		

The results of the mixed-design repeated-measures ANOVA are presented in Table 2. A significant main effect of time was observed (F = 16.086, p < 0.001, ηp^2^ = 0.31), demonstrating a significant increase in brain activity following prosthodontic rehabilitation, irrespective of the treatment modality. In contrast, neither the main effect of the treatment group (F = 0.100, p = 0.905) nor the group × time interaction (F = 0.404, p = 0.671) reached statistical significance, indicating that the magnitude of improvement over time was similar across the three treatment groups.

**Table 2:** Mixed-design repeated-measures analysis of variance (ANOVA) for brain activity changes. * p < 0.05 denotes statistical significance, df: degree of freedom, ηp^2^: Partial eta squared

Source of variation	Sum of squares	df	Mean square	F‑statistic	p‑value	Effect size (ηp^2^)
Group (conventional/coping/attachment)	0.340	2	0.170	0.100	0.905	0.08
Time (baseline vs. two months)	27.232	1	27.232	16.086	<0.001*	0.31
Group × time (interaction)	1.369	2	0.685	0.404	0.671	0.12

Table 3 summarizes the pairwise comparisons of EEG brain activity among the three treatment groups at baseline and after two months of prosthodontic rehabilitation. No statistically significant differences were observed between any pair of treatment groups either before or after the two-month follow-up (all p > 0.05), indicating comparable cortical activity across the three rehabilitation modalities at both evaluation time points.

**Table 3:** Simple main effects of group at each time point (independent samples t-tests). Data are presented as mean difference with 95% CI (confidence interval); Group I: conventional complete denture, Group II: overdenture with coping, Group III: overdenture with attachment; independent samples t-tests were used; p < 0.05 was considered statistically significant; df: degree of freedom.

Time point	Pairwise comparison	Mean difference	t-value	df	p-value	95% CI of difference
Baseline	I vs II	-0.03	-0.110	22	0.913	-0.56 to 0.50
	I vs III	0.21	0.814	22	0.424	-0.31 to 0.73
	II vs III	0.24	0.954	22	0.350	-0.27 to 0.75
Two months	I vs II	-0.29	-0.658	22	0.518	-1.18 to 0.60
	I vs III	-0.46	-1.154	22	0.260	-1.28 to 0.36
	II vs III	-0.17	-0.369	22	0.716	-1.12 to 0.78

The within-group comparisons are presented in Table 4. Significant improvements in EEG brain activity were observed after two months in all three rehabilitation groups. Mean EEG values increased from 8.16 ± 0.68 Hz to 9.08 ± 0.92 Hz in the conventional complete denture group (p = 0.001), from 8.19 ± 0.65 Hz to 9.37 ± 1.22 Hz in the tooth-supported overdenture without attachment group (p = 0.001), and from 7.95 ± 0.58 Hz to 9.54 ± 1.03 Hz in the tooth-supported overdenture with attachment group (p < 0.001). Although the attachment-retained overdenture group demonstrated the greatest numerical improvement in brain activity, between-group differences were not statistically significant.

**Table 4:** Simple main effects of time within each group. Changes in brain activity (Hertz as Hz) within each denture group from baseline to two months (paired t-tests). Data are presented as mean ± standard deviation with 95% confidence intervals. Group I: conventional complete denture (CD); Group II: overdenture (OD) with coping; Group III: overdenture with attachment; CI: confidence interval; *p < 0.05 was considered statistically significant.

Group	Baseline (Hz)	After two months (Hz)	Paired differences	95% CI of the difference		t-value	p-value
	Mean ± SD	Mean ± SD		Lower	Upper		
Conventional CD	8.16 ± 0.68	9.08 ± 0.92	-0.91 ± 0.73	-1.382	-0.450	-4.33	0.001*
OD with coping	8.19 ± 0.65	9.37 ± 1.22	-1.20 ± 0.89	-1.774	-0.642	-4.699	0.001*
OD with attachment	7.95 ± 0.58	9.54 ± 1.03	-1.58 ± 0.87	-2.139	-1.027	-6.27	<0.001*

## Discussion

The present prospective observational comparative study evaluated changes in EEG-recorded cortical electrical activity following prosthodontic rehabilitation with conventional complete dentures, tooth-supported overdentures without attachments, and tooth-supported overdentures with stud attachments. The principal findings demonstrated a significant increase in EEG-recorded cortical electrical activity after two months of prosthodontic rehabilitation, irrespective of the treatment modality. Although the tooth-supported overdenture with attachment group exhibited the greatest numerical increase in EEG-recorded cortical electrical activity, no statistically significant differences were observed among the three rehabilitation modalities. These findings suggest that restoration of masticatory function may be associated with changes in cortical electrical activity following prosthodontic rehabilitation, irrespective of the specific type of removable prosthesis.

The baseline demographic characteristics were comparable among the three groups with respect to age and sex, reducing the likelihood that these demographic variables substantially influenced the observed outcomes. Although comparability in age and sex supports the interpretation of the findings, it does not eliminate the possibility of residual confounding inherent to a non-randomized observational study. Given that aging and sex have been associated with differences in cortical electrical activity and cognitive function, comparable baseline demographic characteristics facilitate the interpretation of treatment-related changes while acknowledging that other unmeasured factors may also have influenced the results.

The significant increase in EEG activity following prosthodontic rehabilitation observed in all groups may be explained by the restoration of masticatory efficiency and reestablishment of sensory stimulation from the oral cavity [6,9]. Tooth loss markedly reduces periodontal mechanoreceptor input and alters trigeminal afferent stimulation of the central nervous system [10]. Previous studies have suggested that restoration of occlusal contacts through prosthetic rehabilitation may improve neuromuscular coordination, stimulate trigeminal sensory pathways, and increase cerebral blood flow to cortical regions involved in mastication, learning, and memory [7,11]. However, these mechanisms were not directly evaluated in the present study and are discussed only as potential explanations for the observed changes in EEG-recorded cortical electrical activity. Functional mastication has also been shown to stimulate hippocampal neuronal activity, increase neurotransmitter release, and improve cortical plasticity, thereby contributing to enhanced brain function [12]. The observed increase in the EEG-derived measure following prosthodontic rehabilitation is interpreted as reflecting enhanced cortical electrical activity associated with restoration of masticatory function. However, because EEG provides an indirect measure of neural activity, these findings should not be interpreted as definitive evidence of improved cortical or cognitive function without complementary neuropsychological or functional assessments.

The present findings are consistent with those of the pilot study by Perumal et al. [3], who reported a significant increase in EEG alpha-wave activity following complete denture rehabilitation, suggesting that restoration of masticatory function may be associated with changes in cortical electrical activity. More recently, the systematic review by Sikri et al. [13] concluded that removable dental prostheses are associated with adaptive neurophysiological changes and discussed their potential role in preserving cognitive function in older adults. However, the present study did not directly assess cognitive function or cognitive health. Therefore, the current findings should be interpreted as demonstrating an increase in EEG-recorded cortical electrical activity following prosthodontic rehabilitation rather than evidence of improved cognitive performance or neural function. Collectively, these studies, together with the present findings, suggest that restoration of oral function is associated with changes in cortical electrical activity, although the underlying mechanisms and their clinical significance require further investigation.

Although the attachment-retained overdenture group demonstrated the largest numerical increase, the group × time interaction was not significant, indicating that the magnitude of improvement did not differ significantly among treatment modalities. One possible explanation is the relatively small sample size, which may have limited the statistical power to detect subtle differences among the rehabilitation options. In addition, although all three treatment modalities restored oral function, the present study did not directly evaluate masticatory efficiency, prosthesis retention, bite force, or proprioception [14]. Therefore, any contribution of these factors to the observed EEG changes remains speculative and warrants further investigation. The two-month follow-up period may also have been insufficient to detect long-term neuroplastic adaptations that could potentially differentiate the effects of various prosthetic designs.

The slightly greater improvement observed in attachment-retained overdentures may be explained by their superior retention, stability, and preservation of periodontal proprioception. Retained mandibular canines maintain functional periodontal ligament mechanoreceptors, which continue to transmit afferent sensory impulses through the trigeminal nerve. In addition, stud attachments improve prosthesis stability during mastication, allowing greater bite force generation and more efficient chewing [15]. These factors may enhance the sensory feedback to the cerebral cortex and hippocampus, resulting in greater cortical activation. Nevertheless, because the intergroup differences were not statistically significant, these potential advantages should be interpreted cautiously.

The present findings are consistent with those of previous studies demonstrating that the restoration of masticatory function positively influences brain activity. Krishnamoorthy et al. [16] reported that mastication enhances cerebral blood flow and stimulates the brain regions involved in memory and cognition. Similarly, Onozuka et al. [17] showed that reduced mastication leads to hippocampal degeneration and impaired spatial memory, thereby emphasizing the importance of functional chewing in maintaining neural integrity. Kimoto et al. [18] observed increased regional brain activation following implant-supported overdenture rehabilitation. In agreement with these findings, the significant increase in EEG activity observed across all groups in the present study suggests that restoration of masticatory function, irrespective of prosthesis type, contributes to enhanced cortical activity and may support cognitive health.

The absence of significant differences between conventional complete dentures and tooth-supported overdentures should not be interpreted as evidence of equivalent long-term clinical performance, particularly in view of the relatively small sample size, which may have limited the ability to detect differences between the groups. While EEG activity improved similarly across groups, other clinically relevant outcomes, such as masticatory efficiency, patient satisfaction, oral health-related quality of life, prosthesis retention, and preservation of residual alveolar bone, were not evaluated in the present study. These variables may favor overdenture therapy and should be investigated along with neurophysiological outcomes in future research.

From a clinical perspective, the present findings suggest that timely prosthodontic rehabilitation may provide benefits that extend beyond the restoration of oral function. Improvement in cortical activity following denture treatment supports the concept that rehabilitation of edentulous patients may contribute to the maintenance of cognitive health through the restoration of effective mastication and sensory stimulation. Clinicians should therefore consider early prosthetic rehabilitation not only to improve mastication and quality of life, but also as a potential strategy for promoting healthy brain function in older adults. Tooth-supported overdentures remain an attractive treatment option whenever suitable abutment teeth are available because of their well-established biomechanical and functional advantages despite the absence of statistically superior EEG outcomes in this study.

This study had several limitations. The sample size was relatively small, and the participants were recruited from a single institution, which may limit the generalizability of the findings. The prospective observational study design without randomization may have introduced selection bias and confounding by indication, as treatment allocation was based on clinical presentation rather than random assignment. Brain activity was assessed only by EEG at a single follow-up interval of two months, which primarily reflects early neurophysiological adaptation following prosthodontic rehabilitation. Consequently, the long-term persistence of the observed EEG changes and their relationship with cognitive outcomes could not be evaluated. Furthermore, direct measures of cognitive function, masticatory efficiency, bite force, and patient-reported outcomes were not assessed. Although all prostheses were adjusted to achieve satisfactory clinical retention and stability before the follow-up assessment, objective measurements of denture retention and stability were not performed. Therefore, the independent contribution of prosthesis retention to the observed EEG changes could not be determined. Future multicenter studies with larger sample sizes, longer follow-up periods, objective assessments of denture retention and stability, comprehensive neurocognitive evaluations, standardized treatment allocation or appropriate adjustment for baseline clinical differences, and advanced neurophysiological assessment techniques are warranted to better elucidate the relationship between prosthodontic rehabilitation and the observed EEG changes.

## Conclusions

Within the limitations of this prospective observational study, prosthodontic rehabilitation was associated with a significant short-term increase in EEG-recorded cortical electrical activity in completely edentulous patients after two months, irrespective of the treatment modality. Although tooth-supported overdentures with stud attachments demonstrated the greatest numerical increase, no statistically significant differences were observed among conventional complete dentures, tooth-supported overdentures without attachments, and attachment-retained overdentures. These findings indicate that prosthodontic rehabilitation is associated with short-term changes in EEG-recorded cortical electrical activity following treatment. Further studies with larger sample sizes, longer follow-up periods, and direct assessments of cognitive and functional outcomes are needed to determine the clinical significance of these neurophysiological changes.

## References

[1] Iacob S, Chisnoiu RM, Zaharia A (2025). Correlation between type of edentulism, age, socioeconomic status and general health. J Clin Med.

[2] Emami E, de Souza RF, Kabawat M, Feine JS (2013). The impact of edentulism on oral and general health. Int J Dent.

[3] Perumal P, Chander GN, Anitha KV, Reddy JR, Muthukumar B (2016). Power spectrum density analysis for the influence of complete denture on the brain function of edentulous patients - pilot study. J Adv Prosthodont.

[4] von der Gracht I, Derks A, Haselhuhn K, Wolfart S (2017). EMG correlations of edentulous patients with implant overdentures and fixed dental prostheses compared to conventional complete dentures and dentates: a systematic review and meta-analysis. Clin Oral Implants Res.

[5] Matsuda R, Yoneyama Y, Morokuma M, Ohkubo C (2014). Influence of vertical dimension of occlusion changes on the electroencephalograms of complete denture wearers. J Prosthodont Res.

[6] Morokuma M (2008). Influence of the functional improvement of complete dentures on brain activity. Nihon Hotetsu Shika Gakkai Zasshi.

[7] Banu RF, Veeravalli PT, Kumar VA (2016). Comparative evaluation of changes in brain activity and cognitive function of edentulous patients, with dentures and two-implant supported mandibular overdenture-pilot study. Clin Implant Dent Relat Res.

[8] Soufineyestani M, Dowling D, Khan A (2020). Electroencephalography (EEG) technology applications and available devices. Appl Sci (Basel).

[9] Sakamoto S, Hara T, Kurozumi A (2014). Effect of occlusal rehabilitation on spatial memory and hippocampal neurons after long-term loss of molars in rats. J Oral Rehabil.

[10] Wang X, Hu J, Jiang Q (2022). Tooth Loss-associated mechanisms that negatively affect cognitive function: a systematic review of animal experiments based on occlusal support loss and cognitive impairment. Front Neurosci.

[11] Watamoto T, Egusa H, Mizumori T, Yashiro K, Takada K, Yatani H (2008). Restoration of occlusal and proximal contacts by a single molar crown improves the smoothness of the masticatory movement. J Dent.

[12] Chen H, Iinuma M, Onozuka M, Kubo KY (2015). Chewing maintains hippocampus-dependent cognitive function. Int J Med Sci.

[13] Sikri A, Sikri J, Saroch R, Gill CS, Gupta R, Pathak C (2025). The relationship between removable dental prostheses and brain activity in elderly individuals: systematic review. Rambam Maimonides Med J.

[14] Narita N, Kamiya K, Yamamura K, Kawasaki S, Matsumoto T, Tanaka N (2009). Chewing-related prefrontal cortex activation while wearing partial denture prosthesis: pilot study. J Prosthodont Res.

[15] Wakam R, Benoit A, Mawussi KB, Gorin C (2022). Evaluation of retention, wear, and maintenance of attachment systems for single- or two-implant-retained mandibular overdentures: a systematic review. Materials (Basel).

[16] Krishnamoorthy G, Narayana AI, Balkrishanan D (2018). Mastication as a tool to prevent cognitive dysfunctions. Jpn Dent Sci Rev.

[17] Onozuka M, Watanabe K, Mirbod SM, Ozono S, Nishiyama K, Karasawa N, Nagatsu I (1999). Reduced mastication stimulates impairment of spatial memory and degeneration of hippocampal neurons in aged SAMP8 mice. Brain Res.

[18] Kimoto K, Ono Y, Tachibana A (2011). Chewing-induced regional brain activity in edentulous patients who received mandibular implant-supported overdentures: a preliminary report. J Prosthodont Res.

